# Greener synthesis of nanostructured iron oxide for medical and sustainable agro-environmental benefits

**DOI:** 10.3389/fchem.2022.984218

**Published:** 2022-09-20

**Authors:** Leong Poh Yan, Subash C. B. Gopinath, Sreeramanan Subramaniam, Yeng Chen, Palaniyandi Velusamy, Suresh V. Chinni, Ramachawolran Gobinath, Veeranjaneya Reddy Lebaka

**Affiliations:** ^1^ Faculty of Chemical Engineering & Technology, Universiti Malaysia Perlis, Arau, Perlis, Malaysia; ^2^ Micro System Technology, Centre of Excellence (CoE), Universiti Malaysia Perlis (UniMAP), Arau, Perlis, Malaysia; ^3^ Institute of Nano Electronic Engineering, Universiti Malaysia Perlis, Kangar, Perlis, Malaysia; ^4^ Centre for Chemical Biology (CCB), Universiti Sains Malaysia, Bayan Lepas, Penang, Malaysia; ^5^ School of Biological Sciences, Universiti Sains Malaysia, Georgetown, Penang, Malaysia; ^6^ Department of Oral & Craniofacial Sciences, Faculty of Dentistry, University of Malaya, Kuala Lumpur, Malaysia; ^7^ Research & Development, Sree Balaji Medical College and Hospital (SBMCH)- BIHER, Chennai, Tamil Nadu, India; ^8^ Department of Biochemistry, Faculty of Medicine, Bioscience and Nursing, MAHSA University, Jenjarom, Selangor, Malaysia; ^9^ Department of Periodontics, Saveetha Dental College and Hospitals, Saveetha Institute of Medical and Technical Sciences, Chennai, India; ^10^ Department of Foundation, RCSI & UCD Malaysia Campus, Georgetown, Pulau Pinang, Malaysia; ^11^ Department of Microbiology, Yogi Vemana University, Kadapa, Andhra Pradesh, India

**Keywords:** biosynthesis, metallic nanostructure, plant waste, nanoparticle, biomass

## Abstract

Nanoscale iron oxide-based nanostructures are among the most apparent metallic nanostructures, having great potential and attracting substantial interest due to their unique superparamagnetic properties. The green production of nanostructures has received abundant attention and been actively explored recently because of their various beneficial applications and properties across different fields. The biosynthesis of the nanostructure using green technology by the manipulation of a wide variety of plant materials has been the focus because it is biocompatible, non-toxic, and does not include any harmful substances. Biological methods using agro-wastes under green synthesis have been found to be simple, environmentally friendly, and cost-effective in generating iron oxide-based nanostructures instead of physical and chemical methods. Polysaccharides and biomolecules in agro-wastes could be utilized as stabilizers and reducing agents for the green production of nanostructured iron oxide towards a wide range of benefits. This review discusses the green production of iron oxide-based nanostructures through a simple and eco-friendly method and its potential applications in medical and sustainable agro-environments. This overview provides different ways to expand the usage of iron oxide nanomaterials in different sectors. Further, provided the options to select an appropriate plant towards the specific applications in agriculture and other sectors with the recommended future directions.

## 1 Introduction

The development of nanoscience and nanotechnology is evolving as a promptly developing field, using various applications in science, research, and technology for the purpose of manufacturing new nanoscale levels of macromolecular matter (Cortez-Jugo et al., 2021; Qindeel et al., 2021). Nanotechnology is quickly developing nanomaterials that can be applied in various commercial products, including bio-sensing paints, sunscreens, cosmetics, clothing, the food industry, and medical devices (Parhi et al., 2020; Epps and Abolhasani, 2021; Huguet-Casquero et al., 2021). A nanostructure can be defined as a system of intermediate size that is between a molecular structure and a microscopic image, at typical dimensions between 1 and 100 nm. A nanostructured material consists of crystallites of nanoscale size with different crystallographic orientations. This material consists of atoms or molecules that become the essential building blocks of materials arranged in nano-sized groups. The composition and building blocks in three-dimensional arrangements can affect the properties of the materials. All natural structures are built up from molecular precursors and substructures of nano-size (Gleiter, 2000).

Recently, nanotechnology has recognized great potential, and nanomaterials are extensively used in a wide variety of modern life—including in the modern agricultural sector—to transform various agricultural practices (Usman et al., 2020). Nanomaterials used in agricultural development are newer ideas that have been proven to recover soil quality, improve the degradation of pesticide residues, and encourage seed germination (Rui et al., 2016). Therefore, the consumption of cheap and abundant agricultural waste has become more pressing than ever. However, the production of such waste is expected to increase rapidly due to the tremendous growth of activities in the modern agricultural sector, which can cause adverse effects to the environment. Therefore, precision farming is the best alternative; it allows precise control at the nanometer scale and reduces production costs through the advancement of nanotechnology. Different types of nanoparticles/nanomaterials have been produced by employing varied approaches and these nanomaterials are with a single type or as hybrid (Ghosh et al., 2020; Chandraker et al., 2022; Kumar et al., 2022; Tamang et al., 2022). Among the generated nanomaterials, silver nanoparticle become one of the prominent metals due to its easier and simple generation from the precursor (Jalab et al., 2021). Silver nanoparticle has different potentials compared to iron oxide nanoparticles (IONP) and other nanoparticles. Similar to silver nanoparticle, IONP is also cheaper and easier to prepare, however, IONPs have totally different property such as, magnetism, which makes them with unique applications. With the magnetic properties, IONPs have been considered in several applications compared to gold, copper, silica, titanium, zinc and so on. With the same properties IONP made the special applications with other metals as a hybrid.

**FIGURE 1:**
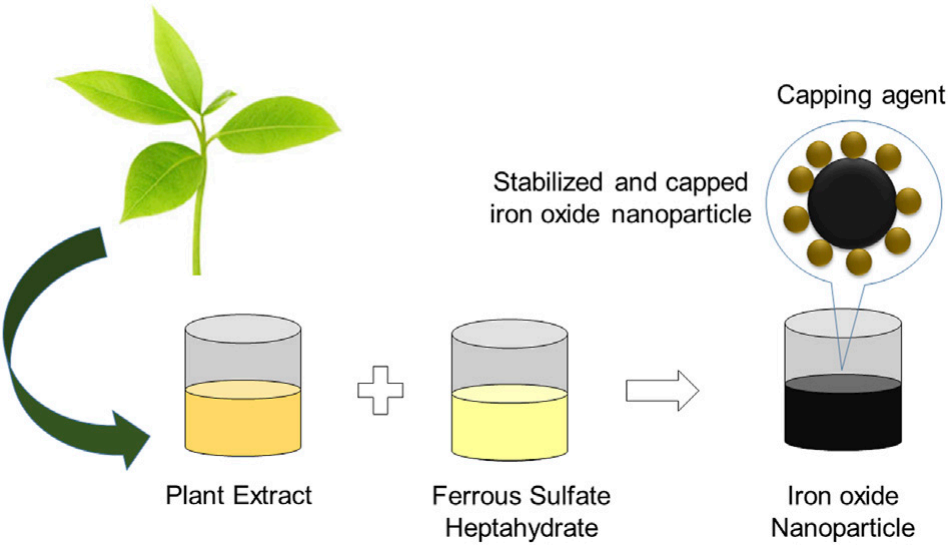
The steps involved in the production of iron oxide nanoparticles (IONP).

**FIGURE 2:**
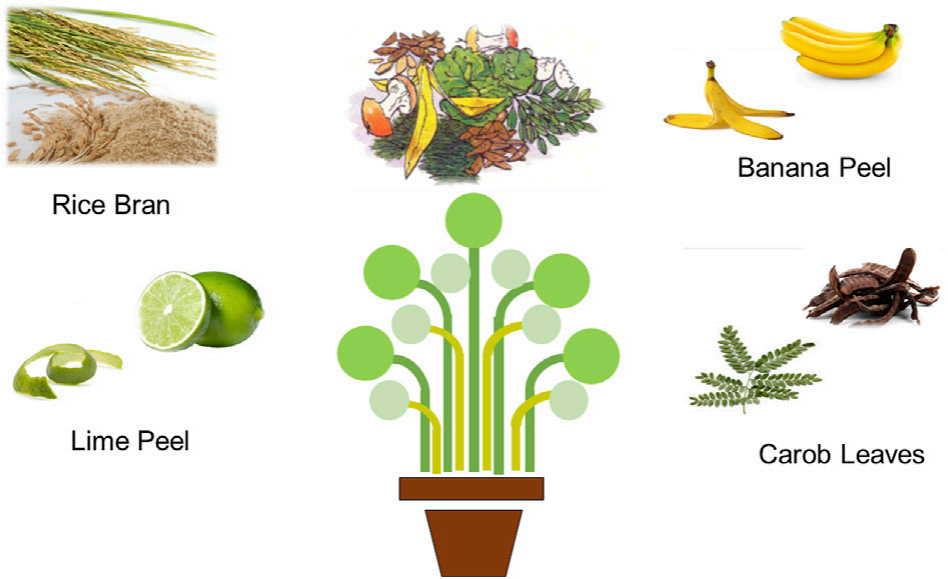
Visual observations of different agro-wastes, which can be used for the production of IONP.

The green production of nanostructures using agro-wastes could minimize the usage of hazardous chemical reagents and reduce the cost of synthesis (Ahmed and Mustafa, 2020; Alkhalaf et al., 2020; Jain et al., 2021). Green methods have been followed widely due to their appealing advantages primarily as cheaper, easier to follow and suitable for larger scale preparation (Behravan et al., 2019; Vanlalveni et al., 2021; Chandraker et al., 2022). Plant and the associated materials are widely used for the production of metallic nanomaterials in different shapes. Because medicinal phytoextract contains alkaloids, phenolics, terpenoids, proteins, lipids, carbohydrates, and other nitrogen-containing chemicals, it provides a route that can be an efficient reductant to reduce precursor into the nanomaterial. The reduced nanomaterial produced by employing medicinal phytoextract as reductant compounds can then be used in biomedical applications because phytoextract is pure and non-toxic by nature. Naturally occurring polyphenols and biomolecules found in plant material wastes act as driving forces and play an active role in the formation of nanostructures with distinct sizes and shapes that are safe, greener, and environmentally friendly (Figure 1) (Herlekar et al., 2014; Lingamdinne et al., 2019; Sahu et al., 2021). Agro-waste is commonly known as agricultural wastes and is defined as the residues in the form of solids, liquids, or slurries that are produced from a wide variety of agriculture activities. These wastes are usually produced from the processing of raw agricultural products through farming activities, including vegetables, fruits, daily products, crops, and meats (Figure 2). Two types of constituents that can be found in agro-wastes are soluble constituents, such as organic acids, and insoluble constituents, such as lignin and cellulose. Furthermore, the major quantity of agro-wastes are comprised of food processing wastes, crop wastes, vegetable wastes, and animal wastes. The composition of the waste depends on the type of agricultural activity and the systems it uses. Moreover, agro-wastes provide an advantage, as the decaying part of plants can be used by farmers to advance the fertility of soil. Agro-wastes are the non-product outputs from the processing of agricultural products that are usually discarded to the environment. Disposal of these wastes generated from agricultural production activities may cause pollution to the environment. Furthermore, some research has proven that agricultural waste can be economically transformed into different nanostructure materials, such as nanowires, nanosensors, nanotubes, and nanofibers, which have gained much attention and are usable in many potential applications due to their unique optical properties, small size, and great surface-to-volume ratio (Mukhopadhyay, 2014).

Moreover, the quality of modern agricultural practices could be enhanced by nanotechnology to generate safer and better agricultural products. There are significant applications in the management of water quality from nanotechnology, such as the removal of heavy metals from pollution to ensure good water quality [5, 6]. Hence, reuse of agro-wastes has a high chance of developing the green production of iron oxide-based nanostructures in different forms, which have unique properties and composition due to their abundance, low cost, and ease of attainability.

## 2 Forms of iron oxide

### 2.1 Magnetite

Magnetite with the chemical formula Fe_3_O_4_ is a mineral iron ore, which is also known as one of the members of the oxide of iron. It is the most common magnetic mineral found in metamorphic, sedimentary, and igneous rocks with black or brownish- black color (Jamlos and MF, 2017). Magnetite is a particle that shows a difference from other iron oxides due to its structure, which has ferromagnetic properties. These properties can offer powerful magnetization of IONP (Smuleac et al., 2011). It also has a face-centered cubic structure on 30 O^2−^ ions by the arrangement of cubic close-packed in a regular pattern. Furthermore, magnetite revealed four different crystalline polymorphs. It also presents both bivalent and trivalent iron ions at room temperature. Magnetite has a crystal structure that belongs to the inverse spinel formed by stacking plans as a polyhedral model (Wu et al., 2015).

### 2.2 Maghemite

Maghemite, γ-Fe_2_O_3_ nanoparticle is a ferromagnetic mineral that has an identical lattice arrangement with magnetite, whereas the iron atoms are in an oxidation state of iron (III). It can be transformed to hematite, which is antiferromagnetic at higher temperatures. Maghemite has a crystal structure of spinel that is like magnetite and has the same electron diffraction configurations (Shokrollahi, 2017). The difference between both oxides is their vacancies in cation sublattice. It can give a high magnetic response and is easily magnetized while reacting to an external magnetic field. The lattice vacancies of maghemite are able to give a stronger magnetization than the other iron (III) oxides, such as hematite. Moreover, the outstanding magnetic properties and less cause of hazard to health have proved that maghemite has received great attention for its biomedical applications. Thus, maghemite has a favorable selection for the production of magnetic nanoparticles for biomedical applications because its structure didn’t cause any significant side effects to humans (Winsett et al., 2019).

### 2.3 Hematite

Hematite (α-Fe_2_O_3_) is an oxide that exists in nature as mineral; it is one of the most common polymorphs and among the most well-known of iron oxides that occur widely in soils and rocks. Hematite has weak ferromagnetic behavior at room temperature; meanwhile, it is paramagnetic when at above temperature of 956 K. It is extremely stable under environmental conditions, so it is easier to synthesize than other oxides. Its crystal structure shows both rhombohedral and corundum types. Furthermore, hematite has received great attention in numerous applications, such as magnetic storage media, gas sensors, and environmental treatments, as it is biodegradable, nontoxic, has low corrosion, and does not require high processing expenses (Wu et al., 2015).

## 3 Greener syntheses of nanomaterials

The reduction between the precursor ion and natural reducing agents—such as plants and microorganisms that consist of a wide variety of species of bacteria, algae, yeast, and fungi—is known as a green synthesis of the iron oxide-based nanostructure (Mahdavi et al., 2013; Saranya et al., 2017; Ikram et al., 2021). There is no use of chemical reagents or organic solvents in green production. This biological production of iron oxide-based nanostructures is proposed as an eco-friendly and cost-effective method, as the reducing agents used are bio-based (Wang et al., 2014). Examples of bio-based agents include plants and their extracts, carbohydrates, peptides, and proteins. A green chemistry approach that connects nanotechnology with plants is known as the plant-mediated production of metal-based nanostructures. Plants and their extracts seem to be the best options for biological reducing and capping agents that are suitable for the green production of nanostructured iron oxide (Parveen et al., 2016). Xing et al. (2014) demonstrated that there are probable mechanisms for the green production of iron oxide-based nanostructures: enzymatic and non-enzymatic reductions. Enzymatic reduction requires biological reducing agents, which are peptides, proteins, and carbohydrates. On the other hand, plants and microorganisms also undergo non-enzymatic reduction to synthesize iron oxide-based nanostructures. Enzymatic reduction is a slower process than non-enzymatic reduction. Non-enzymatic reduction is often a fast process that can be completed within a few minutes and has the ability to handle extreme parameters, such as high temperatures and pH (Ramanathan and Gopinath, 2017).

In the chemical method, surfactants, protective agents, and reducing agents are used to synthesize high-stability NPs and avoid particle agglomeration. There are disadvantages in the chemical methods for the synthesis of iron oxide-based nanostructures, including that the contamination of nanoparticles will occur when different chemicals are used during nanoparticle synthesis. Therefore, two ways of chemical reduction of iron oxide-based nanostructure discovered which uses a strong reducing agent and the weak one as a reducing agent in order to fix the complications.

On the other hand, green synthesis has numerous advantages, including that it is a biocompatible, cost-effective, and environmentally friendly method that eliminates the usage of toxic reagents [15, 16, 17]. It can also be known as a simple method that is able to generate highly stable and well-characterized iron oxide-based nanostructures. The size, structure, and morphologies of the iron oxide-based nanostructure are controlled by altering situations such as the temperature, pH, exposure time, mixing speed, and concentration of the substrate. Hence, green synthesis is an efficient method due to its ability to synthesize iron oxide-based nanostructures by using biological reducing and stabilizing agents.

### 3.1 Green production of nanostructured iron oxide using agro-wastes

The biological production of metal-based nanostructures is known as a green technology from the manipulation of various agro-wastes because it is biocompatible, non-toxic, and does not include any harmful substances (Lakshmnarayanan et al., 2021; Periakaruppan et al., 2021). The green production of nanostructured iron oxide uses agro-wastes without including any toxic chemical reagents, so this method can be said to be more appealing than physical and chemical methods (Prodan et al., 2013; Ansari et al., 2016). This type of synthesis has received great attention and has been actively explored recently. Organic compounds such as flavonoids, polyphenols, and vitamins are significant compounds that can be found in agro-wastes. The reduction of metal precursors can occur from the wide variety of functional groups present in the compounds. These compounds enable agro-wastes to act as reducing or stabilizing agents. Previous studies have shown that the application of food waste has the potential to be utilized for the green production of different NPs (Sharma et al., 2015). The compositions of agro-wastes not only can affect particle distribution but also can affect the size and shape of particles (Mohamad et al., 2013). A previous study reported that mango peel extract can be used for the production of gold NPs as a natural reducing agent. The synthesized gold NPs produced were monodispersed and proved that there was no involvement of biological toxicity in the normal kidney cells of African green monkeys and normal human fetal lung fibroblast cells (Yang et al., 2014). In addition, tangerine peel extract can be used in order to synthesize iron oxide-based nanostructures for the removal of cadmium in contaminated water. The report showed that different concentrations of tangerine peel influenced the size of the particles. The average size of the iron oxide-based nanostructure decreased as the concentration of the tangerine peel extract increased (Ehrampoush et al., 2015). Moreover, the fruit seed waste extract of *Syzygium cumini* as a reducing agent could reduce iron ions to crystalline iron oxide NPs. *S. cumini* seed has a lot of biomolecules and polyphenol compounds for the synthesis of iron oxide NPs. The synthesized nanostructured iron oxides are spherically shaped (Venkateswarlu et al., 2014). The seed extract of *Hordeum vulgare has* the potential to synthesize crystalline nanostructured iron oxide. The biomolecules present in this plant are citric or oxalic acids, which act as reducing gent to facilitate the conversion of iron ions to nanoparticles in aqueous solutions (Makarov et al., 2014). *Citrus limetta* (Mosambi peel) and *Curcuma longa L*. (Turmeric leaves) from agro-wastes in India were found to successfully synthesize nanostructured iron oxide. High polyphenol content was determined from both plant materials, which can be reduced by the metal ions to form complex. These agro-wastes can potentially be used for the biological synthesis of nanostructured iron oxide, which provides great potential for municipal wastewater treatment (Herlekar et al., 2015). A previous study reported that carob leaf extract could be used for the synthesis of nanostructured iron oxide as a natural reducing agent. The synthesized IONPs produced had an average size of 4–8 nm (Awwad et al., 2013). Plant extract of *Clitoria ternatea* (white Clitoria flower) is used to synthesize IONP with the presence of phenolic compounds as a natural reducing agent (Baby et al., 2017). The formation of bonding between phytochemicals in plant extracts and nanostructured iron oxide can produce stable metallic nanoparticles. Furthermore, *Mimosa pudica* root extract contains mimosine, which can act as a reducing agent of iron ions to produce highly stable and crystalline nanostructured iron oxide (Niraimathee et al., 2016a). Furthermore, in another study, *Eucalyptus globulus* leaf extract showed great potential for synthesizing high-density nanostructured iron oxide, since it has significant promise for antimicrobial activities against food-spoiling microorganisms and higher medicinal properties in these plants (Balamurugan et al., 2014). Moreover, a spherical shape and uniformly distributed nanostructured iron oxide with an average size of 33 nm can be produced using the leaf extract of *Caricaya papaya* (Latha and Gowri, 2014). The leaf extract of *Cynometra ramiflora* can be used as a reducing agent to reduce iron ions to crystalline nanostructured iron oxide. The nanoparticles have highly effective antimicrobial activity against *S. epidermidis and E. coli* (Groiss et al., 2017). Table 1 displays the list of agro-waste extracts used for the production of nanostructured iron oxide.

**TABLE 1:** Types of agro-waste extracts used to produce nanostructured iron oxide.

Agro-waste materials	Types of extracts	Size (nm)	References
*Hordeum vulgare*	Seed extract	10–40	Makarov et al. (2014)
*Syzygium cumini*	Seed extract	20	Venkateswarlu et al. (2014)
Banana	Peel extract	10–25	Thakur and Karak, (2014)
Plantain	Peel extract	<50	Venkateswarlu et al. (2013)
*Punica granatum*	Peel extract	—	Irshad et al. (2017)
Tangerine	Peel extract	50	Ehrampoush et al. (2015)
*Zea mays L.*	Silky hairs of corn	84.81	Patra and Baek (2017)
*Brassica rapa L. subs. pekinensis*	Outer leaves of Chinese cabbage	48.91	Patra and Baek (2017)
*Citrus limetta*	Mosambi peel	338.2–488.1	Herlekar et al. (2015)
*Curcuma longa L*.	Turmeric leaves	176.8–685.6	Herlekar et al. (2015)
*Caricaya papaya*	Leaf extract	33	Latha and Gowri, (2014)
Carob	Leaf extract	8	Awwad and Salem (2012)
*Clitoria ternatea*	Leaf extract	73	Baby et al. (2017)
*Cynometra ramiflora*	Leaf extract	—	Groiss et al. (2017)
*Eucalyptus globulus*	Leaf extract	100	Balamurugan et al. (2014)
*Mansoa alliacea*	Leaf extract	18.22	Prasad (2016)
*Ocimum sanctum*	Leaf extract	47	Madheswaran, (2014)
*Rumex acetosa*	Leaf extract	10–40	Makarov et al. (2014)
*Mimosa pudica*	Root extract	67	Niraimathee et al. (2016a)
*Areca catechu*	Shell	50	Vaanamudan et al. (2019)
*Saccharum officinarum*	Bagasse	20–50	Alves et al. (2012)

## 4 Iron oxide nanoparticles

Nanoparticles are particles with nanoscale measurement in the range of 1–100 nm that have received great attention because of their unique small size and great surface-to-volume ratio (Bao et al., 2009; Lee et al., 2018). Nanoparticle (NP) synthesis has great potential for procedures to manage both organic and inorganic toxic pollutants. Nanoscale IONPs are among the most apparent metallic nanoparticles; they have great potential and have attracted substantial interest due to their various specific magnetic behaviors and properties, such as high magnetic perceptivity and unique superparamagnetic traits. This type of nanoparticle is also less sensitive to oxidation and possesses strong ferrimagnetic behavior (Kandpal et al., 2014). Iron oxide is a common chemical compound that occurs abundantly in nature. Iron oxides are magnetic nanomaterials composed of iron and oxygen. Different magnetic properties, structures, and crystal structures are present in iron oxide. Furthermore, there are 16 iron oxides, hydroxides, and oxide-hydroxides. Iron oxide-based nanostructures involve three different phases: magnetite, maghemite, and hematite (Campos et al., 2015).

Recently, iron oxide-based nanostructures have been actively explored due to their various applications in wastewater treatment, cell separation, as contrast agents for magnetic resonance imaging (MRI), for drug delivery to a desired targeted cell, and for hyperthermia and cancer therapies (Irshad et al., 2017; Soetaert et al., 2020; Nguyen et al., 2021). The explorations are expected to further reform the practice of medicine and may have significant applications in all aspects of disease, diagnosis, prevention, and treatment. Speranza et al. (2010) revealed that the advantages are that iron oxide-based nanostructures can be injected into the human body without any significant side effects on health. On the other hand, nanomaterials have been used to produce NPs due to their many applications, which have been revealed by their physical, chemical, or biological characteristics (Zhao et al., 2014; Gaillet and Rouanet, 2015; Chung et al., 2016). Recently, iron oxide-based nanostructures have proven appealing for the elimination of heavy metal pollution because of their magnetic property, small size, and large surface area (Rasheed and Meera, 2016; BhartiJangwan et al., 2022). It has been proven that the synthesis of iron oxide-based nanostructures with high potential applications is in great demand (Ramimoghadam et al., 2014). NPs of magnetic iron oxides, including magnetite and maghemite, are known for their non-toxicity and biocompatibility due to the presence of iron ions that can be used in medicine (Ali et al., 2016). This type of nanoparticle is potentially of high concern to researchers in bio-applications, data storage, and catalysis. Furthermore, the most common biomedical application is magnetic resonance imaging, which enables researchers to improve bio-conjugated magnetic iron oxides concerning brain tumors with simultaneous monitoring (Ali et al., 2016). However, controlling size, shape, stability, and dispersibility could be a challenge for this application (Cantillon-Murphy et al., 2010). An appropriate surface coating, which is emerging with several adequate protection strategies, is significant for controlling the strength of IONPs (Wu et al., 2008). Iron oxide-based nanostructures have a huge surface-to-volume ratio and require high surface energies. Therefore, the surface energies that are able to reduce from the aggregation occur (Wu et al., 2015). IONPs have some disadvantages with naked iron oxide-based nanostructures; therefore, iron oxide-based nanostructures should be stabilized to prevent agglomeration, which keeps the particles from being oxidized and reduces the surface area. Thus, it is important to synthesize iron oxide-based nanostructures with nontoxic biocompatible protective layers to stabilize the magnetism and morphology of IONPs (Tran et al., 2011).

Several methods involved in the synthesis of iron oxide-based nanostructures have been considerably demonstrated (Batool et al., 2021). Physical methods require a very large quantity of energy for evaporation and condensation in a large tube furnace. Parameters in the tube furnace require a longer timeframe to complete the iron oxide NP synthesis (Ramanathan and Gopinath, 2017). In the chemical method, the strong reducing agents in the chemical synthesis of iron oxide-based nanostructures will generate larger sized NPs—though there still remains the challenge of managing the size variations (Rasheed and Meera, 2016). To fix these complications, two ways of chemically reducing iron oxide-based nanostructures have been developed, the first of which uses a strong reducing agent and the other of which uses a weak reducing agent. By using the reduction method, a small size and shape of stable iron oxide-based nanostructures are generated. Among the available methods, green synthesis has been found to be a simple, environmentally friendly, and cost-effective way to generate a stable IONP (Ahmed et al., 2016).

## 5 Potential applications of nanostructured iron oxide

**FIGURE 3:**
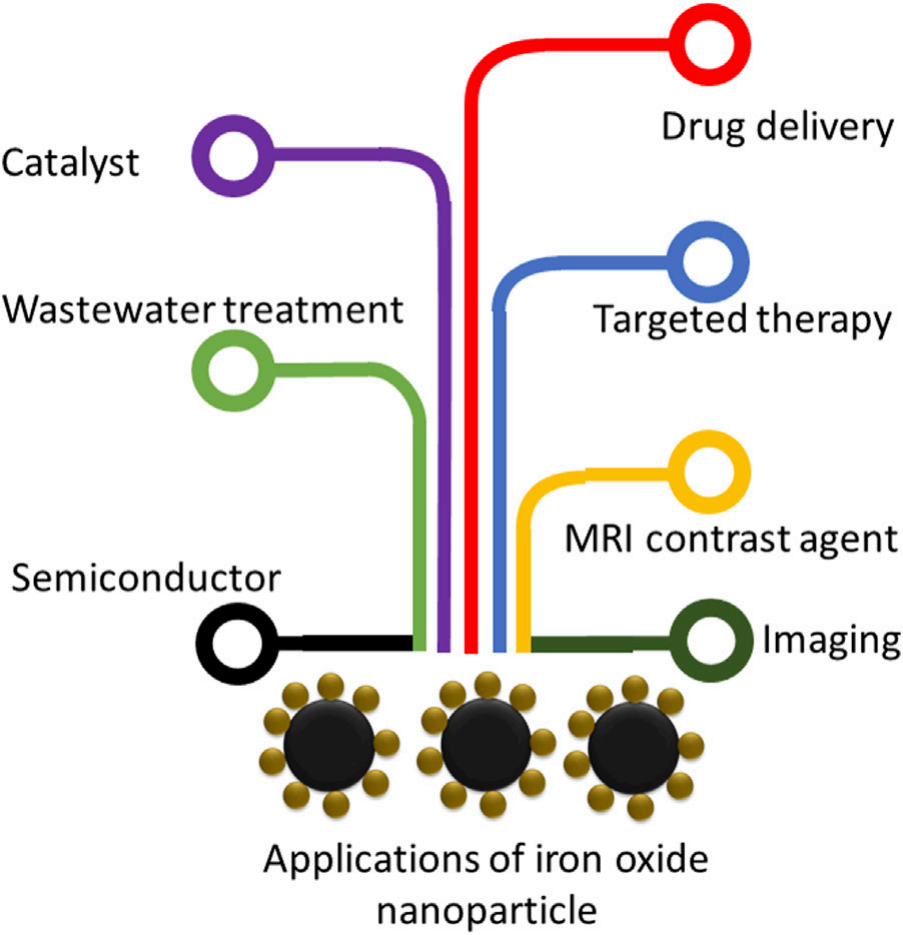
Potential applications of IONPs. A wide range of practical applications are indicated.

The production of IONPs has elevated their importance in several fields, including agriculture, wastewater treatment, catalysts, drug treatment, and imaging (Schneider et al., 2022) (Figure 3).

### 5.1 In the agriculture sector

Agriculture has proven to have potential for various promising nanotechnology applications (Chhipa and Joshi, 2016). The use of nanotechnology in agriculture has increased due to the realization that the productivity of farms would not increase by using conventional techniques. Degradation of soil quality and increased use of fertilizer would lead to necessity of greater energy inputs to maintain the yield by using traditional agricultural practices. Hence, important attention is being paid to the use of nanotechnology in the agricultural sector, as it has the potential to transform food production and conventional agriculture practices. The required amount of farm inputs, such as fertilizers, could be determined by the development of nanomaterial mediated nanosensors that indicate the water or nutrient status of the farm’s products (Ditta et al., 2015). The application of nanobiosensors and other smart delivery systems could be used to attack crop microorganisms in the agricultural sector and offer a capable means of allocating fertilizers and pesticides. The application of nanotechnology in different fields, such as nanofarming and nanofood packaging, has emphasized the ecological balance of its properties (Mukhopadhyay, 2014). It is necessary to determine the optimum levels of IONP to be used with these applications before they are applied.

#### 5.1.1 As a nano-fertilizer

The quality and food production could be enhanced by using fertilizers after the fertilizer responsive varieties and introduction of high yielding. Excess use of chemical fertilizers to increase crop production is not an appropriate option, as they can decrease soil fertility and disturb the soil mineral balance. It can cause air pollution, as residual minerals may leach down into the soil. Excessive use of chemical fertilizers in mineral cycles, soil structures, and food chains across the ecosystem will also lead to inherited changes in future generations of consumers. Therefore, the development of nanofertilizers would be a great option and a new idea for reducing the consumption of excessive fertilizers for crop fields. The application of nanofertilizers also plays a significant role in agriculture because they are able to provide better yields, efficiency in controlling nutrition deficiency, and the environmental boon to nature of reducing soil pollution (Rameshaiah et al., 2015). Furthermore, the yield and growth could be enhanced by nanofertilizers that can provide one or more nutrients to the plants, which eases the better performance of conventional fertilizers that would not supply crops with nutrients directly. Furthermore, the ability for high reactivity and greater absorbance, which is able to improve the growth of crops, has become one of the potential contributions of nanofertilizers. Research has proven the efficiency of nanostructured iron oxide as a fertilizer, which is better than conventional fertilizer, in attaining sustainable growth in agricultural applications with negligible environmental impact. Nanocompounds are able to be absorbed completely and rapidly by plants, as well as to fix nutrient shortages and growing needs with the potential contribution of nanofertilizers (Rui et al., 2016). Apart from this, the ratio of IONP usage to chemical fertilizer needs to be determined to make a better plant yield.

#### 5.1.2 In high-performance of nanosensor

Nanosensors have been developed that have proven to be user friendly and able to control the level of soil nutrients. This technology can reduce the consumption of fertilizer and minimize pollution to the environment. It is also beneficial in precision agriculture, which can be used to detect weeds and pesticides (Chhipa and Joshi, 2016). Molecular interactions in biological media can be detected by magnetic nanosensors, which exhibit biocompatibility and high specificity. This causes variations in the spin-spin relaxation times of adjoining water molecules upon target-induced nanoassembly development. There are various molecular targets alternating from enzymatic activity, proteins, specific mRNA, and pathogens; those with a low sensitivity in the femtomole range (0.5–30 fmol) can be detected by these magnetic nanosensors (Perez et al., 2004). For high-performance analysis with nanosensors, the size of the IONP might play a crucial role and vary with different sensing surfaces and sensing systems. For applications in the agricultural sector, the IONP-mediated high-performance can be used to monitor the abundance of important biomarkers, which are responsible for fruit ripening, accumulation of heavy metals in rice grains, the occurrence the pollutant/detrimental compounds, and so on.

#### 5.1.3 Photocatalysis

Photocatalysis involves a catalyst, which also employed with nanotechnology, that requires light and has been developed to increase the reactions involved. It is defined as a material that is utilized for transformation and regenerates its composition after each cycle of relations, which is accomplished by producing electron-hole pairs and absorbed light. Iron oxide is one type of metal oxides that is utilized for light absorption to induce a charge separation process for oxidizing organic substrates. Some research has shown that the anions and radicals generated from the photocatalytic activity of metal oxides could be used as photocatalysts that react with pollutants to reduce them to non-harmful byproducts, including in the agriculture sector. Photocatalytic nanomaterials have been used for the degradation of pollutants and heavy metals; these compounds are detrimental to agricultural products. Therefore, this type of catalyst has great potential for hydrogen production and environmental remediation associated with agriculture (Khan et al., 2015). It was attested that waste materials can be destroyed by this technique under catalysts with light irradiation (Khedr et al., 2009). Similar to the applications with photocatalysis, IONP is also highly associated with electrocatalysis and is well-versed with electrochemical reactions as an electrode interface, in which IONP is rolling to be an electron acceptor/donor and a catalyst. This application has been widely seen in dielectric sensing systems, where the involvement of the IONP accelerates the dipole moment. Dipole moment with IONP speeds up ionic movement and enhances the output signal or display in several folds. Due to the involvement of dipole moment in a wide range of applications including on the sensing surfaces, the involvement of IONP is highly appreciated (Saber et al., 2022). Further, the ionic/molecular vibrations with IONP regarding to the above applications have potential future benefits in different industrial sectors (Ali Noman et al., 2021; Gabelica et al., 2021). The involvement of photocatalysis with IONP is highly beneficial for the purpose disinfection in water and air matrices (Gao and Keller, 2021).

### 5.2 Types of nanomaterials

#### 5.2.1 Nanowire

Nanowires can be defined as nanostructures that have a diameter or thickness less than or equal to 100 nm. A nanowire can have the properties of an insulator, a semiconductor, or a metal, depending on the composition of the materials. Some of these materials include carbon, silicon, germanium, and a wide variety of metals, such as copper, iron, and gold. These nanostructures hold great interest and potential for both scientific and technical communities due to their capability for greatly reduced device dimensions. Furthermore, nanowires can be utilized for many possible applications, such as electronic devices, optics, and the sensing of proteins and chemicals using semiconductor wires. This small nanostructure has the potential for important advances in computer science because it is a good conductor through which electrons easily pass. Moreover, artificial protein-coding DNA can be created using amino acid nanowires. This advanced technique could be used to facilitate the creation or production of proteins, which could potentially lead to advances in therapeutic applications. A report has shown that nitrilotriacetic acid can be used as a chelating agent to produce iron oxide semiconductor nanowires under an efficient synthesis technique to produce polymeric chains with heat treatment. These nanowires exhibited unique magnetic properties and showed great sensitivities to acetic acid gases and ethanol (Wang et al., 2008).

#### 5.2.2 Nanofiber

Nanofibers have emerged as fibers with a range of nanometer-sized for downstream applications. They exist as one-dimensional nanomaterials for a wide variety of research and have great potential for many advanced applications. The methods of production and the types of polymers used are factors that can affect the diameter of nanofibers. These materials are unique due to their ability to possess extremely great surface-area-to-volume ratios and appreciable mechanical strength (Kenry and Lim, 2017). Moreover, nanofibers can also be created from two types of polymers: natural and synthetic. Natural polymers are often identical to macromolecular substances present in the human body. Examples of natural polymers are cellulose, collagen, gelatin, silk, chitosan, and keratin. Meanwhile, many synthetic polymers are utilized to form nanofibers; they represent the largest class of biomaterials. These involved polyurethane, polycaprolactone, and polylactic acid. Presently, there are different systems for the production of nanofibers, including self-assembly, electrospinning, and phase separation. Among these, electrospinning has emerged as the most used and attractive technique for the production of polymeric biomaterials into nanofibers. This method has the capability to control the size and composition of ultrathin fibers with a simple and straightforward experimental setup along with the involvement of IONP. Furthermore, nanofibers are able to provide many possible applications that are used in drug delivery, tissue engineering, and cancer diagnosis (Vasita and Katti, 2006).

## 6 Biomedical applications

### 6.1 Drug delivery

**FIGURE 4:**
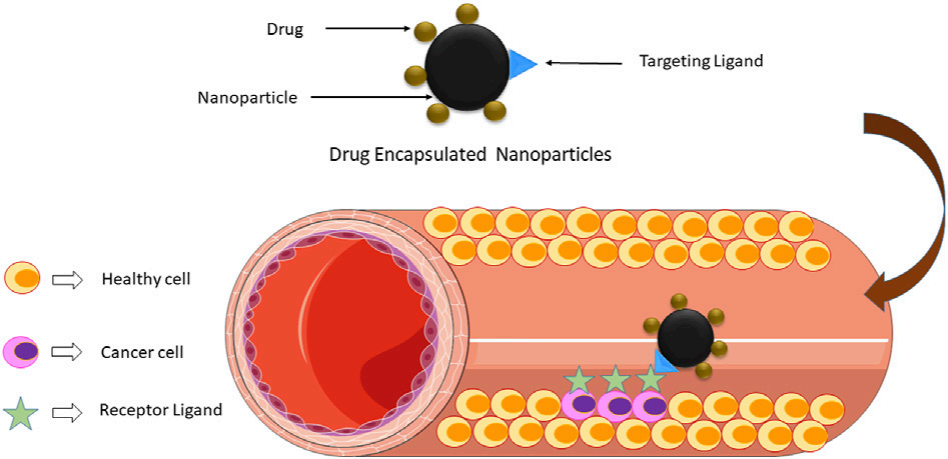
Encapsulation: The process in drug-encapsulated particles to clear infected cells.

**FIGURE 5:**
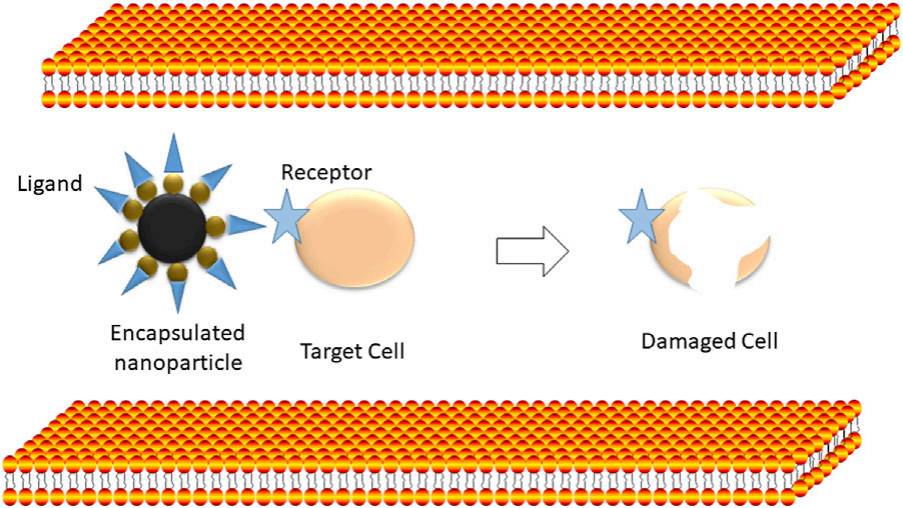
Targeted drug delivery: the selective targeting of cancer cells.

The conception of drug delivery using magnetic nanoparticles was proposed as one of the current technologies that can give birth to a new era in cancer therapy. Magnetic nanostructures, such as iron oxide-based nanoparticles, act as drug delivery agents. They can be attached to a drug to be delivered at the cancer site under the effect of an external magnetic field (magnetic drug delivery; Figures 4, 5). The dosage of drugs required can be reduced, and side effects can be eliminated by attaching drugs to specific sites (Faraji et al., 2010). Mody et al. (2014) demonstrated that the surfaces of iron oxide-based nanostructures have magnetic cores with an external coating of inorganic metals or organic polymers, which can be functionalized to make them biocompatible through the attachment of numerous bioactive molecules. After that, a catheter is used to inject the delivery drug agent into the bloodstream to place the injection site near the target. In this context, the selection of an appropriate probe molecule to reach the target site is crucial.

### 6.2 Hyperthermia

The use of synthesized nanostructured iron oxide for magnetic hyperthermia has attracted considerable attention as an effective therapy for cancer treatment. The superparamagnetic iron oxide-based nanostructure can convert magnetic energy from an external high-frequency field to particles in heat form. The range of temperature used is normally 41–47°C for the heating of cells. The particles begin to vibrate and produce heat to destroy pathogenic microbes or cancer cells within the magnetic field. The heat generated can be used in an *in vivo* application to raise the tumor tissue’s temperature (Thoidingjam and Tiku, 2017). The mechanisms that can be utilized by heating are frictional heating prompted by the relationship between the iron oxide-based nanostructure and the medium and eddy current heating that occurs from an alternating pulsed magnetic field (Abenojar et al., 2016). Furthermore, tumor cells are more sensitive to a rise in temperature compared with healthy ones. The advancement in temperature optimization may enhance radiation therapy and chemotherapy (Giustini et al., 2010). Recently, numerous modern techniques have required hyperthermia, which has been developed for the heating treatment of cancers (Wu et al., 2015). Yanase et al. (1998) demonstrated that the application of magnetite cationic liposomal nanoparticles, which have positive surface charges, is efficient for hyperthermia treatment in cancer therapy. Moreover, there is a significant challenge to increasing the heating rates of iron oxide-based nanostructures that require reaching therapeutic temperatures for this application. Increasing heating rates can be an alternative approach to increasing the monodispersity of iron oxide-based nanostructures. As one study showed, the hyperthermia effect of superparamagnetic iron oxide-based nanostructures can be enhanced dramatically with a coating of gold. It is possible for the application of very low rate oscillating magnetic fields from the results revealed (Mohammad et al., 2010).

### 6.3 Magnetic resonance imaging

**FIGURE 6:**
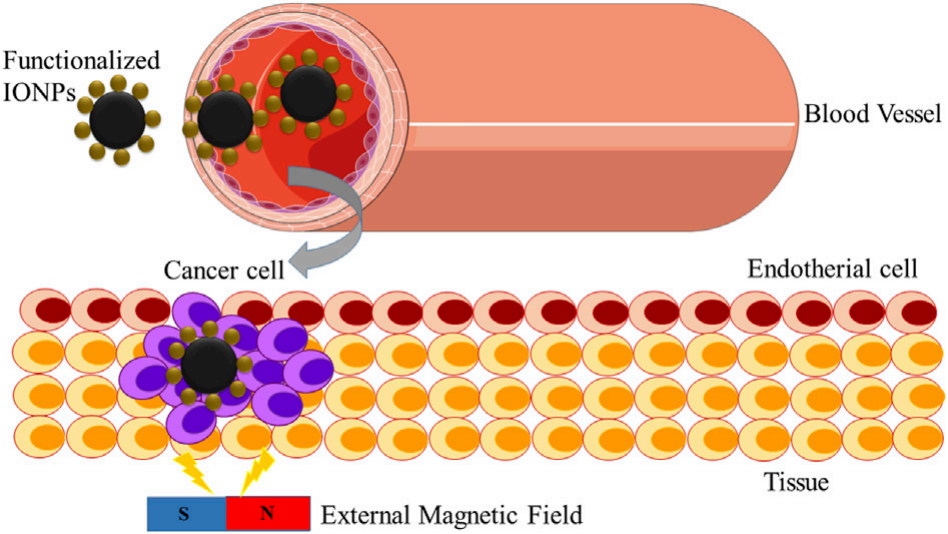
The basic steps of IONP-mediated MRI.

Tumors form in humans across a series of procedures that occur over an extended period. Accretion of genetic variations will lead to advanced alteration of a normal cell into a malicious cell during this process (Gupta et al., 2010)**.** MRI is an imaging system used principally in medical applications to create high-quality, detailed pictures of the human body in radiology. A strong magnetic field, radio waves, and an electric field gradient are utilized by MRI scanners to generate images of parts of the body. This application does not involve ultrasound, X-rays, or CT scans (Hanson, 2009). Moreover, this system can enable us to see inside cartilage, joints, ligaments, and muscles to identify various sports injuries. MRI is also able to observe internal body structures and analyze several disorders, such as tumors. It is widely used in research to measure brain structure and function, among other things (Faraji et al., 2010). Various types of antibodies directed to various kinds of receptors can be used to couple on iron oxide-based nanostructures. After that, the tumor can bind with the specific sites of the particles. The targeted site can be delivered to an iron oxide-based nanostructure using an external magnetic field. Furthermore, cell tracking with a resolution that approaches the size of the cell is acceptable when the cell overloads the magnetic iron oxide-based nanostructure (Figure 6). The monitoring of cell therapy and the observation of biological processes can be provided from *in vivo* cell labeling in MRI (Wu et al., 2015).

### 6.4 Bioseparation

Bioseparation is one of the most important applications in the synthesis of IONP. Superparamagnetic colloids are ultimate for this technique due to the nature of their magnetization, which assists in the passage of biomaterials with a magnetic field. Basically, the separation of specific biological entities is often involved in this application in biomedical research. This application is usually involved in cell, bacteria, virus, enzyme, protein, gene, protein, and *in vitro* DNA separation. Magnetic bioseparation has various advantages, including the ability to retrieve or localize with a magnet more quickly than traditional separation techniques. Magnetic iron oxide-based nanostructures have various characteristics for this application, as they have a high surface area and a small size. They are also more cost-effective and faster than traditional column affinity chromatography. Bioseparation is generally enhanced by surface-functionalized magnetic iron oxide-based nanostructures with suitable intermediate efficiency and alteration of polymers and surfactants for introducing functional end groups (Wu et al., 2015). Li et al. (2011) investigated how carboxymethylated dextran-coated magnetic iron oxide-based nanostructures can be synthesized using the co-precipitation method for bioseparation application. The particles covered with antibodies provide the ability for the antigen to separate from the sample solution, as they have superparamagnetic properties.

## 7 Environmental applications

Recently, many researchers have been attracted to the environmental applications of magnetic iron oxide-based nanostructures. These nanoparticles have been proven to have great potential for the removal of both organic and inorganic pollutants. Regardless of some still unresolved uncertainties related to the application of iron oxide-based nanostructures, the synthesized iron oxide-based nanostructures are predictable as beneficial tools in the rectification of a wide variety of pollutants in water, soil, and air at both experimental and field levels. Moreover, iron oxide-based nanostructures exhibit great flexibility for *in situ* applications. Modified iron oxide-based nanostructures that support and catalyze nanoparticles have been synthesized to further improve the speed and efficacy of remediation nanoparticles (Faraji et al., 2010). Tran et al. (2011) demonstrated that an iron oxide-based nanostructure can serve as a talented adsorbent for the removal of toxic metals, such as lead and arsenic, in contaminated water. The applications of synthesized iron oxide-based nanostructures are further expanded for different fields, and they have been revealed [85,86].

## 8 Antimicrobial activity of synthesized iron oxide nanoparticles

Antimicrobial activity can be identified by various classes of microorganisms. It is also known as the minimum inhibitory concentration, which is the lowest concentration of an antimicrobial agent that prevents the growth of bacteria after overnight incubation [87]. Four small filter paper discs were inoculated with the plant extract, solvent, antibiotics, and the synthesized IONP and then placed on the prepared agar plates of indicator organisms. The plates were incubated at 35–37°C for 24 h. The zone of bacterial inhibition could be investigated on agar plates after the incubation period. The zone of clearance around each filter paper showed that the antimicrobial activity of the specimens occurred on the agar plates. There are some factors that might affect the interpretation and results of antimicrobial activity: the incubation conditions, the concentration of the filter disc, and the agar plate of the indicator organism (Ramanathan and Gopinath, 2017). A report revealed that erythromycin as an antimicrobial agent was analyzed with an iron oxide-based nanostructure, and its antimicrobial characteristics were investigated against the bacterial culture of *S. pneumoniae* [88]. For antimicrobial applications with IONP, the condition to be optimized for the desired ratio as it causes the major output [88,89]. The biomedical applications of iron oxide nanoparticles have been further expanded with antimicrobial potential against a wide range of microbes, anti-tumour and cancer therapies (Sangaiya and Jayaprakash, 2018; Table 2).

**TABLE 2:** Size-based iron oxide nanoparticle mediated biomedical applications.

Size (nm)	Methods followed	Inhibition/cell arresting
0–20	Precipitation, co-precipitation, laser ablation, thermal decomposition, laser pyrolysis, ultra centrifugation	*Staphylococcus aureus, E. coli*, MCF-7, Violamycine B1, HepG2, PC3, PC12
20–50	Co-precipitation, biosynthesis, lipid film rehydration, green synthesis, chemical precipitation, thermal decomposition, laser ablation, Penners & Koopal method	*Proteus mirabilis*, *E. coli*, Hek293 cell, MCF-7, HBL-100, A549, cancer therapy, MRC-5, Neuro2A, Human placenta
50–100	Direct heating, chemical precipitation, ultra sonication, sonication	*Aeromonas hydrophilia*, *E. coli*, HER2-PA, human alveolar epithelial cell line

## 9 Future perspectives

**FIGURE 7:**
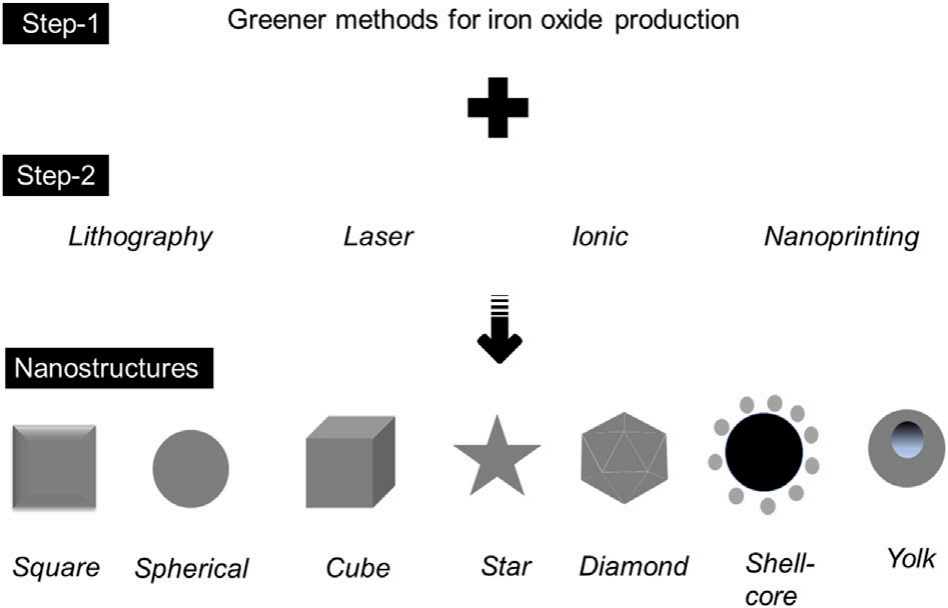
The production of different iron oxide-based nanostructures, including the involvement of other methods (step 2) along with greener production (step 1).

As discussed in this review, biological methods, especially for recycling agro-wastes, are found to be highly potential and reliable. Direct reduction of precursors under ambient conditions is classic and can be performed in low-resource laboratories. The capping of compounds from plants seems promising, and it can be scaled up in an easier way. For scaling-up purposes, for instance, with the production point in the cosmetics industry, determining the optimum conditions, such as temperature, reaction time, and usage of the right precursor, are mandatory. In addition, analyzing the primary compounds from plants is mandatory, and they bring a great increment in the final reduced product. Apart from the standard agro-wastes mediated by green synthesis, the implementation of methods generated for the production of IONP needs to be considered [6–8, 14]. Along with the methods discussed in this review, the involvement of other physical and chemical methods in greener ways might be associated. These directions will pave the way for making IONP in different nanostructures. In general, the production of nanoparticles under the routine classic method is spherical in shape, but the size may vary depending on the desired condition(s). However, with several current practical necessities, it demands different nanostructures, primarily for expanding surface areas. Surface expansion by modifying the nanostructure is greatly implemented in biomolecular capturing, particularly for bionanosensor development. The crucial part of generating different nanostructures is the right additional method. The popular methods include lithography, nanoprinting, exposure by laser, and ionic implantation (Figure 7).

Compared to other organic nanoparticles, iron oxide nanoparticles have several practical advantages, such as readily forming oxides for the ultimate use in surface functionalization. Glutaraldehyde is the most common cross-linking reagent because it is easily accessible for linking two amines. On the IONP, glutaraldehyde can be functionalized through any amine coupling, such as 3-(aminopropyl)triethoxysilane. The use of glutaraldehyde as a multifunctional reagent can be reduced by drastic modification with an aminated probe during the immobilization process. Another cross-linking option for IONP with biomolecules is between amine and carboxyl on the substrate or receptor. The reaction between amine and carboxyl can be activated followed by stabilization with 1-Ethyl-3-(3-dimethylaminopropyl) carbodiimide and N-hydroxy succinimide-mediated coupling reactions. Both amine-carboxyl coupling and glutaraldehyde modifications on IONP are highly feasible. Iron oxides with nanomaterials have the potential to garner ideal surface functionalization, as oxides are reliable for making simple chemical reactions with linkers. Enhancement of oxides or making the oxide surface is achieved by treating the surface with potassium hydroxide. Iron nanomaterials for biomolecular immobilization are greatly utilizing the above functional groups, and the presence of different functional groups on the surface expands the applications. Further, iron oxides are highly in use with commercial cosmetic sectors and as ore, catalysts, pigment, and thermite. As revealed elsewhere, iron oxide nanoparticles are highly magnetic, which is not so with other nanomaterials. Current investigations brought several insights with the generation greener, heterostructured and hybrid materials, may help for further expansions (Chandraker et al., 2022; Sahu et al., 2022).

## 10 Conclusion

The review gleaned important aspects on the production of iron oxide nanoparticle and the associated downstream applications. This paper highlights the environmentally benign manufacture of iron oxide-based nanostructures using a straightforward process, as well as their uses in sustainable agriculture and medical fields. This overview offers numerous suggestions for increasing the use of iron oxide nanoparticles in various fields. Additionally, options were given to choose a suitable plant for the applications in agriculture and other sectors, along with suggested future developments along the green production. The green production of nanostructures has received abundant attention and has been actively explored recently because of their various beneficial applications and properties. The biological production of the nanostructure is known as green technology due to the manipulation of various plant materials and because it is biocompatible, non-toxic, and does not include any harmful substances. However, need to consider the presence of toxic or physiologically irrelevant compounds in some plant species. Based on our review, the green synthesis of iron oxide-based nanostructures under biological methods using plant extracts was found to be the preferable method due to its environmentally friendly, simple, non-toxic, and cost-effective manufacturing of iron oxide-based nanostructures. Polysaccharides and biomolecules in plant extracts are used as stabilizers and reducing agents for the green production of iron oxide-based nanostructures. The production of high-efficiency iron oxide-based nanostructures has attracted several researchers for the study of nanoparticles because of their small size and large surface-to-volume ratio. This dimensional appearance provides unique properties to nanoparticles. The size, shape and dimension of the produced nanomaterials may be affected by the level of compounds extracted from the plant source. The synthesis of iron oxide-based nanostructures by physical and chemical methods is unfavorable compared with biological methods because of the usage of chemical reagents, the extreme consumption of energy that requires equipment, and the longer time to complete nanoparticle synthesis. These factors lead to the formation of contaminated and hazardous nanostructured iron oxides. Moreover, this type of nanoparticle potentially invites great interest for researchers in bio-applications, data storage, and catalysis. Iron oxide-based nanostructures can act as antimicrobial agents, as their antimicrobial properties can be further explored in the biomedical industry. Furthermore, iron oxide-based nanostructures have created a demand for the elimination of pollutants in wastewater treatment. Hence, the synthesis of iron oxide-based nanostructures by an eco-friendly method using plant extracts as a reducing and capping agent has great potential for the production of large-scale iron oxide-based nanostructures. Considering other side, using green production with a large scale it is necessary to be optimized for the conditions to be used. In addition, the optimal condition may vary with the abundance of the capping and reducing agents.
